# τFCS: Multi-Method Global Analysis Enhances Resolution and Sensitivity in Fluorescence Fluctuation Measurements

**DOI:** 10.1371/journal.pone.0090456

**Published:** 2014-02-28

**Authors:** Neil R. Anthony, Keith M. Berland

**Affiliations:** Department of Physics, Emory University, Atlanta, Georgia, United States of America; University of California, Irvine, United States of America

## Abstract

Fluorescence fluctuation methods have become invaluable research tools for characterizing the molecular-level physical and chemical properties of complex systems, such as molecular concentrations, dynamics, and the stoichiometry of molecular interactions. However, information recovery *via* curve fitting analysis of fluctuation data is complicated by limited resolution and challenges associated with identifying accurate fit models. We introduce a new approach to fluorescence fluctuation spectroscopy that couples multi-modal fluorescence measurements with multi-modal global curve fitting analysis. This approach yields dramatically enhanced resolution and fitting model discrimination capabilities in fluctuation measurements. The resolution enhancement allows the concentration of a secondary species to be accurately measured even when it constitutes only a few percent of the molecules within a sample mixture, an important new capability that will allow accurate measurements of molecular concentrations and interaction stoichiometry of minor sample species that can be functionally important but difficult to measure experimentally. We demonstrate this capability using τFCS, a new fluctuation method which uses simultaneous global analysis of fluorescence correlation spectroscopy and fluorescence lifetime data, and show that τFCS can accurately recover the concentrations, diffusion coefficients, lifetimes, and molecular brightness values for a two component mixture over a wide range of relative concentrations.

## Introduction

Modern scientific studies increasingly demand accurate characterization of the spatial and temporal dynamics of specifically identifiable molecules [1]–[3]. Fluorescence fluctuation spectroscopy (FFS) methods have thus become important research tools as they enable detailed investigations of the chemical and physical properties of molecules or molecular systems in a variety of complex environments [4]–[9]. When FFS data is analyzed successfully, impressive resolution of sample composition and dynamics is often achievable. This includes the unique capabilities to measure dynamics over a wide range of time-scales, to accurately measure molecular concentrations, and to directly measure the stoichiometric composition of interacting molecular species. On the other hand, there are a number of fundamental challenges that can limit the overall capabilities of FFS methods, notably, limited resolution, parameter stability during curve fitting, and problems with fitting model verification. For example, the second component of a two component sample can be challenging or impossible to resolve if its concentration or molecular brightness is significantly lower than the concentration or brightness of the primary species. Also, in typical FCS measurements, it is generally not possible to resolve two separate sample components by diffusion analysis unless their diffusions coefficients differ by a factor of approximately two [10]. In addition, FCS measurements offer limited capability to discriminate between fitting models when knowledge of the sample composition or physical dynamics driving fluctuations is not available *a priori*, as is often the case for measurements within living cells or other complex systems. These types of limitations can leave detection of a large number of potentially important molecular phenomena and interactions outside current experimental capabilities. A variety of strategies have been implemented to overcome some of these experimental limitations, including multi-color FCS measurements and the development of numerous molecular brightness based statistical analysis approaches [5], [11]–[21]. In addition to specific advances in fluctuation methods, other approaches have also proved useful for enhancing experimental resolution in fluorescence measurements. Simultaneous analysis of the multiple spectral signatures acquired using multi-parameter fluorescence detection (MFD) [22]–[25] can greatly reduce false assumptions that commonly occur at the resolution limits of the single techniques alone [26]. Global analysis [27]–[33], i.e. curve fitting using global parameters ‘linked’ across multiple data sets, greatly constrains the fitting parameter space that can fit all experimental data simultaneously, enhancing resolution, and also improving model discrimination capabilities in curve fitting routines.

Here we introduce a new conceptual approach to fluorescence fluctuation microscopy that leverages the strengths of fluctuation measurements, MFD, and global analysis, and significantly enhances the broad capabilities of fluorescence fluctuation measurements. Specifically, we apply simultaneous acquisition of fluorescence correlation spectroscopy (FCS) and fluorescence lifetime data and use multi-modal global analysis to analyze both data types simultaneously with common linked fundamental parameters, an approach we refer to as τFCS. We demonstrate that τFCS is remarkably successful in fully resolving the physical properties of a two-component mixture for cases where previously reported FFS methods would be unable to accurately determine the sample composition and dynamics. We also discuss how this new analysis approach improves model discrimination capabilities and improves parameter stability in curve fitting procedures. We note that FCS and lifetime measurements have previously been implemented together [34], [35], although the τFCS approach is fundamentally different from the lifetime-filter based FLCS method and has significantly less demanding requirements for the measurement signal statistics, an important practical advantage. Moreover, the fundamental strategy of combining multi-parameter fluorescence acquisition with global analysis is easily extended to other fluorescence measurement modes (e.g. anisotropy, FRET) with minimal theoretical modifications, and should thus be widely applicable in most experimental systems where FFS measurements are used.

## Theory

Simultaneous global fitting of both lifetime and FCS data requires that the theory for each be written in terms of common linkable parameters. Traditionally, lifetime theory is written in terms of the excited state lifetime values and fractional intensities of each molecular species, with no direct link to the concentration or molecular brightness values that are used in FFS theory. We thus introduce a complete theory, derived from basic fluorescence principles, describing fluorescence lifetime decay histograms in terms of the molecular concentrations and molecular brightness parameters used in FFS. Fluorescence lifetime measurements require pulsed laser excitation sources, and thus τFCS theory describes pulsed laser excitation, spans picosecond to second time scales, and incorporates effects due to finite fluorescence lifetimes [36]–[40]. To completely describe fluorescence dynamics under pulsed excitation in terms of common fluorescence parameters we must consider both micro- and macro-time scales. Micro-time (ps to ns) is used to describe the excited state dynamics of fluorescent molecules following pulsed excitation, and resets to zero after each incident laser pulse. Macro-time (µs to s) describes longer time scale behavior, such as fluorescence fluctuations due to physical or chemical dynamics, and is continuous from the start of the experiment. The theory presented below is for two-photon laser excitation [37], [38], [40]–[44], as used in the reported experiments, although the details could be easily applied for single photon excitation with only minor modifications.

### Two-photon fluorescence measurements

Considering the micro-time photodynamics of a single molecular species treated as a simple two state model, we previously derived an expression describing the steady state probability, 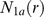, that a molecule will occupy the excited state immediately after a laser pulse passes through the sample [38], [41], [44], with:
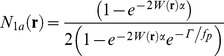
(1)


Here, 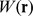 represents two-photon molecular excitation rate [44] for pulsed laser excitation that includes saturation and finite lifetime effects, with a spatial dependence determined by the spatial profile of the focused laser excitation source. The laser pulse repetition rate is 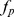, and for simplicity the temporal pulse profile is assumed to have constant amplitude with pulse width 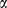, an assumption that has no important consequences for this work. The parameter 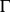 is the spontaneous relaxation rate of excited state molecules. For a molecular concentration 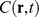, we expect fluorescence emission of 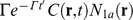 photons per unit volume per unit time following each laser pulse. The measured instantaneous fluorescence signal, 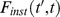, is the integrated signal from the full sample volume given by:
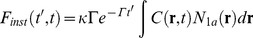
(2)where 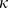 is a sample and equipment dependent constant that incorporates all parameters describing the excitation and optical collection efficiencies, such as absorption cross sections, quantum yields, and detector efficiencies.

Equation (2) contains two distinct time variables that characterize the micro- (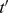) and macro-time (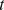) variations in the fluorescence signal. To measure fluorescence lifetimes, we are interested in the micro-time behavior, and in principle, Eq. (2) represents the fluorescence decay curve following each laser pulse. However, in practice, limited photon numbers require that fluorescence lifetime measurements are performed over millions of excitation pulses, with macro-time averaged histograms accumulated to give the micro-time resolved average fluorescence signal:
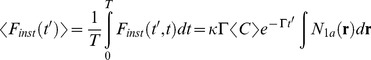
(3)where the total acquisition time, *T*, is sufficiently long that the spatial and temporal concentration fluctuations are averaged out.

Alternatively, for FFS measurements we are primarily interested in the macro-time variations in fluorescence and can instead average out the micro-time behavior. The macro-time averaged fluorescence signal thus depends explicitly on the time dependent concentration and can be written as:
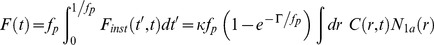
(4)


While 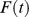 averages out the micro-time dynamics we omit the angular brackets indicating temporal averaging to avoid notational confusion when presenting FCS theory below. One can of course average over both micro- and macro-times to find the average fluorescence intensity:
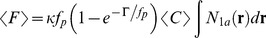
(5)


For purposes of notation consistency with FCS measurements, it is convenient to rewrite the fluorescence signal in terms of an “effective” molecular excitation rate, 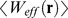, that describes the time-average number of excitation events per second at position **r**. The effective excitation rate has a simple relationship with the average fluorescence intensity given by 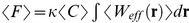 [40], [41]. Comparison with Eq. (5) indicates that 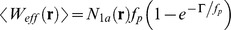. Using this notation, we can rewrite Eqs. (3) and (4) above as:
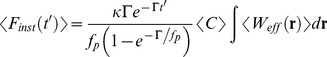
(6)and
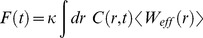
(7)


Writing the fluorescence signals in terms of 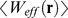 allows for direct comparison with standard FFS notation. In particular, FFS measurements often refer to an “observation volume” [40], [45] which is defined as:
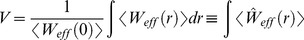
(8)where 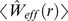 is the normalized fluorescence excitation probability that defines the profile of the observation volume, again including both saturation and finite lifetime effects. Higher order moments of 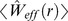 are generally also needed to fully describe FFS theory. For FCS the additional required parameter is called the gamma factor [45]–[47] given by:
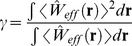
(9)


This parameter characterizes the uniformity of the fluorescence signal from molecules located at various locations within the volume and the effective steepness of the boundary defining the volume. We note that some authors prefer to incorporate the gamma factor into their definition of the volume, defining an effective detection volume as 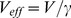 [7], [47], [48].

Using this volume notation, we can write the average fluorescence signal as:
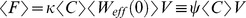
(10)where we have introduced the “molecular brightness” parameter 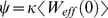, so named because it reports the average number of fluorescence photons measured per molecule per second [12], [13], [49]. We note that molecular brightness is not a fundamental quantity, but depends both molecular properties (cross section and quantum yield), excitation conditions (laser power, pulse width, and beam waist), and the measurement instrumentation (detector and collection optics efficiencies).

Returning to Eqs. (6) and (7) we can write the full time dependence of the fluorescence signals as:
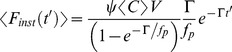
(11)and
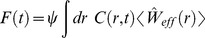
(12)


### Fluorescence Lifetime and FCS Data

Using the above theory it is straight forward to express the data from both lifetime and FCS measurements in terms of common global parameters. For lifetime data, which is composed of histograms from a total of 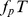 laser pulse cycles, and binned into time channels of width 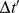, the recorded signal can be written as:
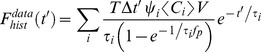
(13)where the subscript 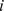
represents each independent fluorescence species within the sample, and emission rates are replaced by fluorescence lifetimes, 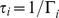. Similarly, using well developed FCS theory [4], [40], [45], [46], [50] the correlation function for a multi-component sample is described as:
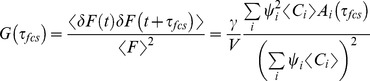
(14)where 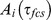 represents the temporal relaxation of the correlation function for species 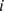. For example, for pure diffusion with diffusion coefficient 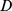 in a three dimensional Gaussian volume with radial beam waist 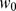, and an axial beam waist 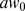:
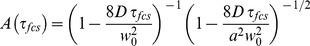
(15)


The factor, *a,* in the axial beam waist is sometimes referred to as a structure parameter in FCS literature. Equations(13), (14) & (15) clearly show how simultaneously acquired lifetime and FCS data sets depend on common global parameters, here concentration and molecular brightness, in addition to unique measurement specific parameters such as their diffusion coefficient and fluorescence lifetime. In fact, the theory is also valid for sequentially acquired lifetime and FCS data provided the sample has not changed between measurement modes, although in practice simultaneously acquisition is the best way to ensure that condition. Subsequent data analysis using these linked global parameters facilitates enhanced model discrimination and constrains fitting parameter space to greatly enhance overall experimental resolution, as demonstrated below. We also note the different functional dependencies on these global parameters, whereby the amplitude of the lifetime data scales with 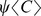 while the amplitude of the FCS data scales with 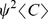. These differences in parameter dependence provide significant constraints for model discrimination in fitting routines.

The theory introduced above accurately describes fluorescence signals originating entirely from the molecular species of interest. In many cases there may be an additional background signal arising from room light, scattered laser light, or background fluorescence from sample contaminants or autofluorescence. If the background signal is significant compared to the signal of interest, then the background must be accounted for to accurately apply τFCS theory. The correction depends on the nature of the background. If the background is not constant, with measurable lifetime or non-trivial fluctuation dynamics, then it must be treated as an additional molecular species according to the theory introduced above. For room light or other time-constant background signals the correction for lifetime data is performed simply by subtracting the background from the total signal, while the correction for FCS requires a corrected correlation function amplitude. For an independently measured background signal, 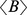, the corrected correlation function amplitude is given by [45], [51]:
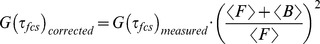
(16)


Here 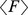 represents the fluorescence signal of interest described in Eq. (10) above, i.e. the measured background signal would need to be subtracted from the measured average signal amplitude, 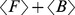, to determine the value of the average fluorescence.

## Methods

### Microscope Setup

Measurements were performed using a home-built two-photon laser-scanning setup built around an inverted microscope (IX71, Olympus) [37], [52]. Pulsed excitation, provided by a Titanium:Sapphire Tsunami laser (∼100 fs pulses at 80 MHz; Spectra Physics) tuned to 800 nm, was introduced to the optical path *via* a dichroic mirror (675DCSX, Chroma Technology) and focused into the sample using an Olympus 60× water objective (UPLSAPO60XW, Olympus; NA  = 1.2). Power was controlled using a λ/2 plate and polarizing cube, and set to 5 mW at the sample. Non-descanned fluorescence signals were measured after a low pass filter (E700SP, Chroma Technology) using a hybrid photodetector (Becker and Hickl HPM 100-40; Boston Electronics). 200 s data points were collected using a time correlated single photon counting (TCSPC) module (PicoHarp300, PicoQuant GmbH). Laser pulse synchronization was provided by a battery powered fast photodiode (New Focus, Model 1621). The TCSPC histograms and autocorrelation curves were calculated using SymPhoTime software and exported for analysis in Igor Pro (Wavemetrics, Inc., OR). TCSPC histograms were binned at 4 ps, and selected to contain approximately 5×10^7^ counts. Autocorrelation curves were calculated from 0.0001 to 100 ms with 16 points per time coarsening (Nsub) using the entire 200 s data set.

### Binary Dye Titrations

Experiments were performed with mixtures of two Rhodamine dyes, selected because their diffusion coefficients are too similar to be resolved *via* standard FCS analysis. Rhodamine 6G (R6G; Sigma Cat# 252433) and Rhodamine B (RhB; Sigma Cat# R6626) dye solutions were prepared in HBS-EP buffer (10 mM Hepes, 150 mM NaCl, 3 mM EDTA, 0.005% Polysorbate 20; TEKnova Cat# H8020), and samples were loaded into 8-well chamber boxes (Lab-Tek II; No. 1.5 coverglass, Nunc; Thermo Fisher). Concentration titrations were performed to obtain a calibrated series of dye mixtures, starting with an 800 µl mixture with R6G and RhB at 50 nM each, a 1∶1 concentration ratio. R6G/RhB concentrations ratios >1 were achieved by sequentially replacing 400 µl of sample volume with an equal volume of 100 nM R6G. Similarly, concentration ratios <1 were prepared by replacing half the sample volume with 100 nM RhB. This procedure provided sample mixtures with concentrations ranging from 4 to 97 nM for R6G and 4 to 89 nM for RhB, leading to a final R6G/RhB concentration ratio ranging from approximately 0.04 to 20.

Data were acquired at each titration step, and the full titration was repeated three times. Error bars reflect the average and standard deviation of these three repeated experiments. To ensure prepared sample concentrations matched their expected values, control measurements were performed in the same sample chamber with solutions of each dye independently (either R6G or RhB), using FCS measurements to determine the actual sample concentration. The same titration procedure described above was repeated, except that HBS-EP buffer or solutions of the same dye being measured were used to replace 400 µl of solution to respectively decrease or increase concentrations. Dye solutions in HBS-EP exhibit stable and reproducible concentration values, provided the chamber boxes were thoroughly cleaned prior to each use. To clean, a single chamber box well was washed with detergent, followed by repeated rinses with hot water, and finally scrubbed with a cotton swab using ultra-pure water. The cleaning procedure was repeated before each titration. Measurements of ultra-pure water in the cleaned chamber never showed signal above the background level of ∼1 kHz, measured independently with the laser out of mode-lock.

### Data Analysis

TCSPC histograms and FCS autocorrelation curves (ACFs) were analyzed using custom and native global analysis routines in Igor Pro. For τFCS analysis, a TCSPC histogram and simultaneously acquired ACF are considered together as one independent τFCS data set (Fig. S1a). All fits in Igor Pro were performed using a Levenberg-Marquardt non-linear least squares algorithm which minimizes the χ^2^ value, for a τFCS data set defined as:
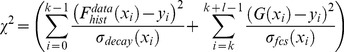
(17)where *k* and *l* are the number of data points in the lifetime histogram and the calculated autocorrelation function respectively. Observation volume parameters, *w_0_* and *a*, were calibrated using R6G in H_2_O solution assuming a diffusion coefficient of 426 µm^2^s^−1^ [53].

We present two different approaches for data analysis. The first is applicable when a τFCS data set is available for only a single sample condition, e.g. a single concentration ratio, which is a common experimental scenario. In this case, the analysis is “global” in that the TCSPC and FCS data have common global fitting parameters (molecular concentration and molecular brightness) that are linked across the data types (each has a single value for both types of data), and other local parameters including the lifetimes and diffusion coefficients. A second analysis approach is used when multiple τFCS data sets are available with one or more experimental variables, such as sample concentration, varied across the data sets (Fig. S1b). For this case, the global fitting routine can be applied to all of the τFCS data sets simultaneously, with a single set of global parameters that are linked across *all* of the τFCS data sets and separate local parameter sets for each τFCS data set. Data fits to experimental decay curves included a temporal offset such that the peak of the instrument response function (IRF) corresponds to 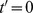, but were not reconvolved with the IRF during fitting. The IRF was recorded using hyper-Rayleigh scattering [54] from colloidal gold solutions (Sigma Cat# G1652) at low excitation powers.

TCSPC histogram data sets were found to contain significant systematic non-Poissonian noise at GHz frequencies (Fig. S2; red line), originating from differential non-linearities within the TCSPC module and other electronic noise. This systematic noise was found to vary linearly with total photon count per time bin in a highly reproducible manner such that this systematic noise could be removed from the signal. A data point of uncorrelated room light containing a comparable or greater number of photon counts as experimental acquisitions was recorded to determine the correction. A 100 point binomial smooth operation was performed to remove Poissonian noise while retaining the lower frequency systematic noise (Fig. S2; black line) [55]. The smoothed reference noise data set was then scaled by the photon counts, yielding a multiplicative factor that effectively removes the non-Poissonian systematic noise from the signal while retaining the inherent Poissonian noise [56]. This procedure was followed for all experimental TCSPC data sets.

### Simulated Data

Simulated data sets were created using Eqs. (2) & (3) for a given parameter set followed by the addition of random noise. TCSPC histograms were created with a total of 5×10^5^ counts followed by the addition of Poissonian noise for a given number of counts per bin. Noise was also added to FCS curves, with noise levels determined using noise levels from experimental FCS curves acquired under comparable conditions [57]. The ACF acquisition time, *T*, for all calculated data sets was 30 seconds. Each χ^2^ surface point is the average of three repetitions with unique simulated noise. All parameters were assumed to be unknown and were not fixed during the analyses, however, we did implement the 〈F〉 constraint (Eq. 10) to remove fitting problems associated with the covariance of the concentration and molecular brightness.

## Results

The primary goal of this work is to demonstrate how experimental resolution and model discrimination capabilities in FFS can be dramatically enhanced by using MFD and multi-method global analysis, here shown through the implementation of τFCS. We thus demonstrate the capability to resolve the molecular composition of a mixture of two identical molecular weight fluorescence dyes, Rhodamine 6G and Rhodamine B, for which standard FCS experiments would be unable to identify the presence of the two sample components (D_R6G_  =  390 µm^2^s^−1^; D_RhB_  =  465 µm^2^s^−1^) [10] or to accurately recover their concentrations and other physical properties since their diffusion coefficients are too close [10]. The fluorescence lifetimes of these two dyes are easily resolved (τ_R6G_  =  3.92 ns and τ_RhB_  =  1.63 ns), and the use of MFD thus provides an important contrast parameter. However, lifetime fitting alone can only resolve lifetime values and fractional intensities and cannot determine molecular concentrations, diffusion coefficients, or molecular brightness values that report on interactions. Even after determining the presence of two species from the lifetime data, a traditional MFD approach fails to recover this information since FCS analysis of data from this mixture can still not achieve a stable fit for a two-component model. On the other hand, τFCS analysis does produce stable fits and accurate parameter recovery, thus providing a vastly improved functionality over FCS and/or lifetime measurements alone.

**Figure 1:**
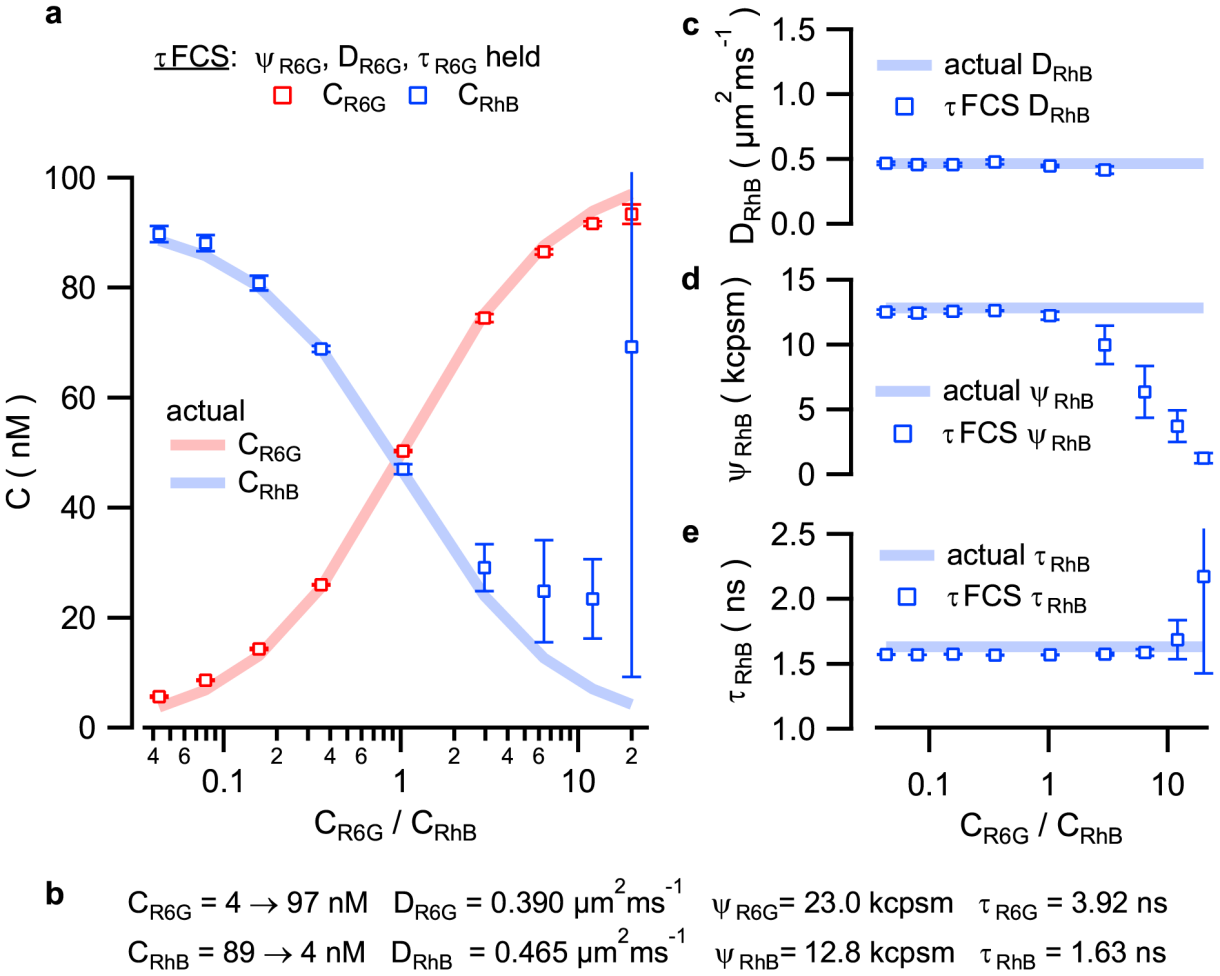
τFCS analysis of binary dye mixtures with known R6G and RhB concentrations. (a) Recovery of molecular concentrations across nine known concentration ratios using τFCS. Measured concentrations are shown as red and blue squares, and the solid lines show the known concentration of each dye mixture. (b) Sample parameters detailing concentrations, diffusion coefficients, molecular brightnesses and fluorescence lifetimes of the prepared R6G and RhB mixtures. Recovery of RhB diffusion coefficient (c), molecular brightness (d), and fluorescence lifetime (e) respectively. Data points and error bars represent the average and standard deviation of three repeated experiments.

To illustrate the strength of this method we analyze nine individual τFCS data sets acquired from rhodamine dye mixtures with RhB and R6G dye concentrations ranging from approximately 3 to 97 nM, providing concentration ratios (C_R6G_/C_RhB_) spanning almost three orders of magnitude. Comparison of τFCS fitting results (Fig. 1a, squares) to the known dye concentrations (Fig. 1a, solid lines) demonstrates accurate recovery of molecular concentrations for each species over a fairly wide range of concentration ratios. This achievement is significant considering that FCS or lifetime measurements alone are not able to recover this information at all. These results, fitted one R6G:RhB concentration ratio at a time, do require some *a priori* knowledge for one of the sample components in order to obtain stable data fits. Here we assume that the lifetime, molecular brightness, and diffusion coefficient of the R6G molecules (τ_R6G_, ψ_R6G_ & D_R6G_) can be measured independently, thus serving as fixed fitting parameters during τFCS analysis (see parameter values in Fig. 1b). We note, however, that prior knowledge of these three parameter values (τ_R6G_, ψ_R6G_ & D_R6G_) is not sufficient to achieve stable fitting results using FCS analysis alone. Only by further assuming prior knowledge of the brightness of the second species can one achieve somewhat stable fits, and even then some of the numerically stable fitting results return inaccurate parameters. Any error in the assumed brightness values also translates directly into errors in the recovered concentrations (Fig. S3). The use of τFCS avoids these problems without any assumptions about the second brightness value, and thus offers a significant advantage.

R6G has a higher molecular brightness than RhB and is the calibrated species in τFCS analysis (fixed values for its diffusion coefficient, lifetime and brightness), and as such, its concentration (Fig. 1a, red squares) can be accurately recovered across all points of the titration. Concentrations of the less bright RhB are harder to measure, yet we still see accurate concentration measurements up to a concentration ratio of approximately 3 (Fig. 1a, blue squares). Above the concentration ratio of approximately 3 the recovered value of C_RhB_ becomes unstable in the curve fitting routines and is sensitive to initial parameter guesses due to the covariance of the molecular brightness and concentration parameters. These instabilities can also been seen in the recovery of the diffusion coefficient (Fig. 1c) and molecular brightness (Fig. 1d) of RhB, which are otherwise quite accurate at lower concentration ratios. This observation is not unexpected since at higher concentration ratios the RhB signal becomes an increasingly small fraction of the total fluorescence signal. Also, since each molecular species' contribution to the measured correlation function amplitudes varies with the square of its molecular brightness, the molecule with lower brightness is harder to measure at lower concentrations than the brighter molecular species. Lifetime measurements alone can accurately determine the fluorescence lifetimes of multiple sample components independently of any FCS analysis, reflected by the measured lifetime values which do not exhibit similar instabilities even at high concentration ratios (Fig. 1e).

It is clear that combing lifetime and FCS data for fitting as a single τFCS data set results in greatly improved capabilities for resolving the molecular composition of a sample. The major limitations of the method as introduced so far are that some independent knowledge of the sample properties (e.g. τ_R6G_, ψ_R6G_ & D_R6G_) was required for stable curve fitting and the RhB concentration was not recoverable at the higher concentration ratios. Each of these limitations can be overcome if one leverages the full power of global analysis. Specifically, if an experimental parameter can be varied over a series of measurements then global analysis allows curve fitting for the entire measurement series with common experimental parameters ‘linked’ across all data sets. For the example introduced here, the concentration ratios of the two dyes varies from one mixture to the next, but other dye properties including the fluorescent lifetimes, diffusion coefficients, and molecular brightness values must share the same values for all mixtures. Full global analysis of the data thus involves simultaneously fitting all of the data sets with a single set of globally linked fitting parameters for the lifetime, molecular brightness, and diffusion coefficient (τ, ψ & D) of each species in the sample. Two additional “local” fitting parameters, C_R6G_ and C_RhB_ are associated with each individual data set to account for the different molecular concentrations of each rhodamine dye for each concentration ratio.

**Figure 2:**
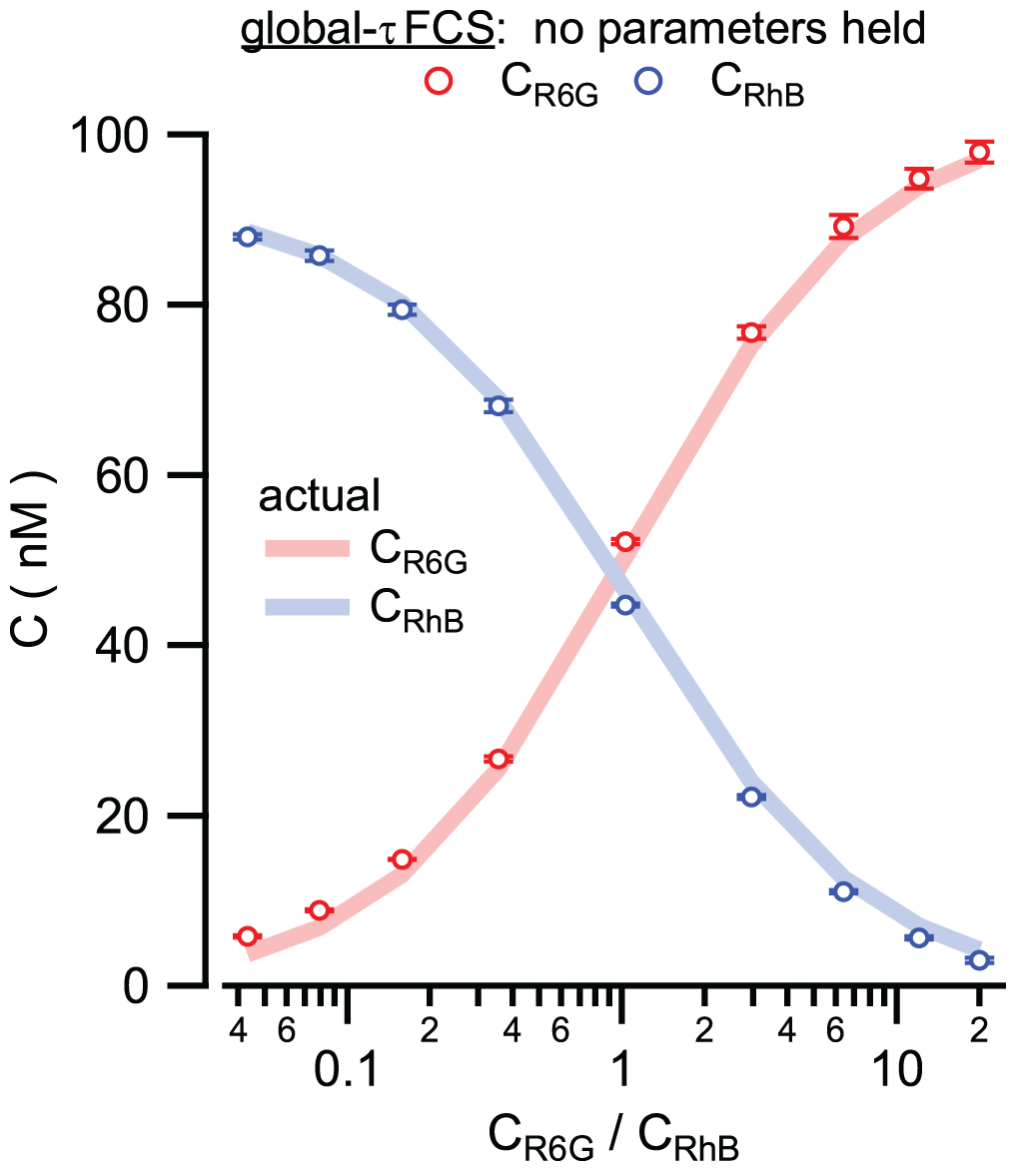
Comparison of Global-τFCS analysis with known R6G and RhB sample mixtures. Recovery of molecular concentrations across nine known concentration ratios using Global-τFCS. Calibrated known concentrations (solid lines) compared to recovered concentrations of R6G (red circles) and RhB (blue circles). Data points and error bars represent the average and standard deviation of three repeated experiments.

The results from the Global-τFCS fit are shown in Fig. 2, and the returned global parameters are shown in Table 1. As can be seen immediately, the full global fits achieve remarkable accuracy across the entire measured concentration range. This includes accurate recovery of the molecular concentration of RhB, the species with lower molecular brightness, even when it constitutes only a few percent of the molecules within the sample. This level of sensitivity for a minor species goes well beyond what has been achievable in fluctuation measurements, demonstrating the greatly enhanced sensitivity and resolution available using the global τFCS approach. Moreover, these extraordinary global fitting results were obtained *without any constraints* on fitting parameters, and unlike the individually fit τFCS data sets the full global fits assume no prior knowledge of molecular brightnesses, diffusion coefficients, lifetime values, or any other parameter for either species. These attributes offer tremendous advantages and flexibility in applications of this method. For complex experimental systems it may be impossible to isolate an individual molecular species for calibration purposes, or calibrations performed under one sample condition (e.g. diffusion coefficients for isolated molecules) may not accurately reflect actual values in a different sample condition (e.g. diffusion coefficients within a living cell). The full global analysis approach completely eliminates the need for any molecule specific calibration measurements, and still returns accurate results across a wide range of sample compositions.

**Table 1:** Molecular parameter values recovered using Global-τFCS compared to known parameter values.

		*Global-*τ*FCS*		*Parameter Value*
		Fit	Rel. Err	
R6G	*D*	0.39±0.01	0.8%	0.39±0.01
	τ	3.91±0.01	0.2%	3.92±0.01
	*ψ*	22.3±0.2	2.9%	23.0±0.70
RhB	*D*	0.46±0.01	0.6%	0.47±0.02
	τ	1.57±0.01	3.7%	1.63±0.01
	*ψ*	12.8±0.1	0.5%	12.8±0.4

The accuracy of the recovered information goes well beyond previous experimental capabilities, which is more remarkable given that no fitting constraints or *a priori* assumptions were required for these fitting results.

We note that while global analysis on its own offers powerful enhancements in many curve fitting applications, the use of global analysis alone does not explain the success of the τFCS approach. In particular, one can attempt to fit the same data shown above using a global FCS fitting approach (no lifetime data), but with only minimal success. Achieving numerically stable fitting results with global FCS (alone) first requires holding fixed the diffusion coefficient and molecular brightness parameters for one species, which is not required for global τFCS, and even then the fit results are highly dependent upon initial guesses for parameter values (see Fig. S4) and can still be unstable. Thus, while one can sometimes get marginally reasonable fits to the data using a global FCS fit (though never close to the accuracy of τFCS), such fits can also return inaccurate results and there is no reliable method to sort the accurate from the inaccurate fit results. The τFCS fits, which leverage the enhanced capabilities of multi-modal global analysis, do not suffer from any such problems and return stable and accurate fitting results every time, independent of initial parameter guesses and without fixed fitting parameters. As with any global analysis methods, the success of this approach depends fundamentally upon there being common linkable parameters across various measurement conditions, i.e. a given molecular species has the same brightness in each sample condition.

**Figure 3:**
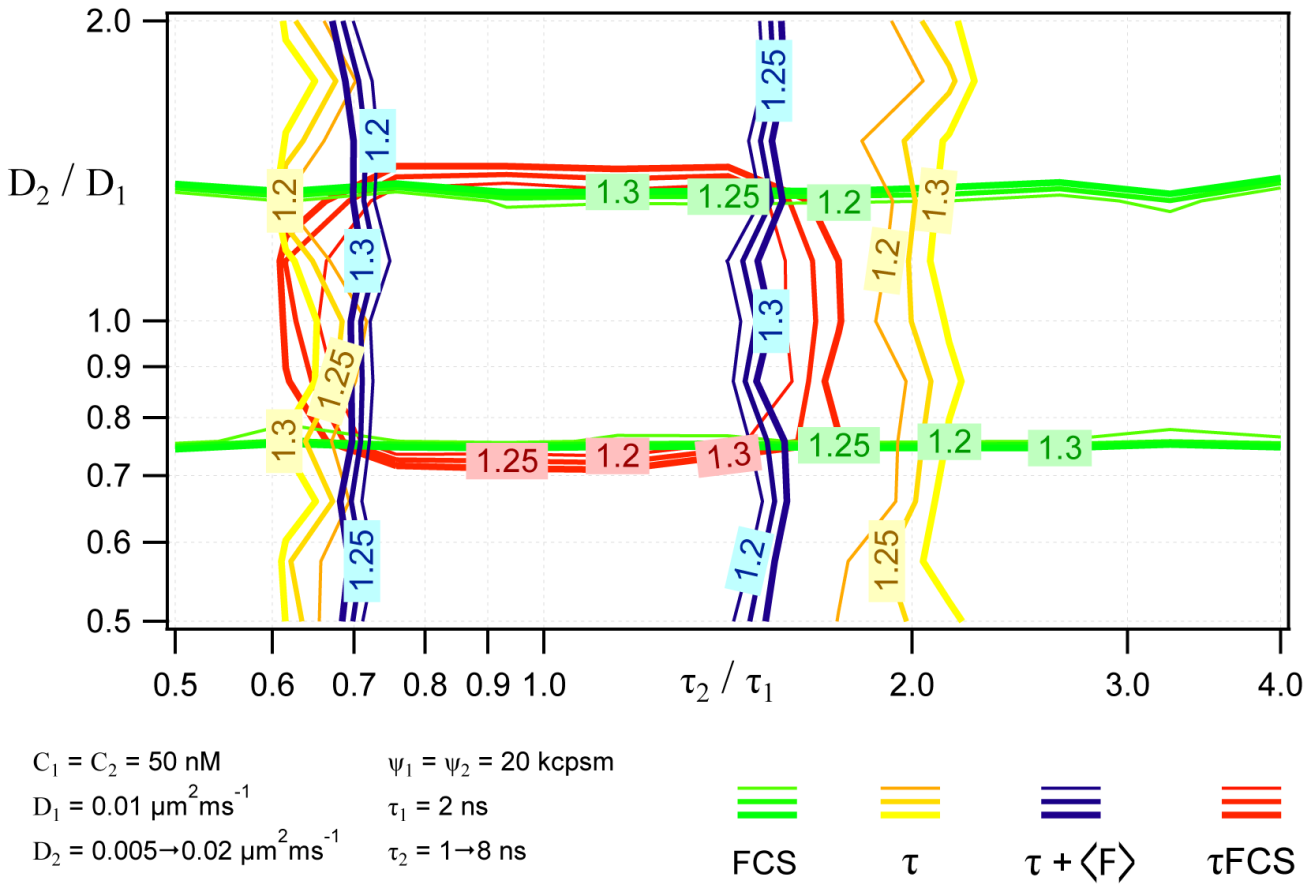
Identifying a second component from simulated mixture data *via* incorrect single component analysis. These curves shown represent the chi-squared contours for the different fitting methods when fitting two-component data with a single component model. When the χ^2^ value exceeds ∼1.3 we conclude that the single component analysis fails, indicating the presence of a second component in the sample. Four χ^2^ surfaces demonstrate the boundary in parameter space for 1) FCS (green lines); 2) Fluorescence Lifetime (yellow lines); 3) Fluorescence Lifetime with average intensity constraint (blue lines); and 4) τFCS with average intensity constraint (red lines).

When correct fitting models are identified, τFCS yields highly accurate curve fitting results, as shown above. Since sample composition is often unknown *a priori*, an important consideration regarding the overall applicability of the τFCS approach is the extent to which curve fitting procedures are able to successfully discriminate between different fitting models. To test this we employed computationally generated data sets for binary mixtures, and fitted data from multi-component samples to an (incorrect) single component model. We then determined the range of lifetime values and diffusion coefficient ratios for which curve fitting to a single species model indicated a sufficiently poor “goodness of fit” to warrant rejecting the single component model. Figure 3 shows these analyses, plotted as cross sections of χ^2^ surfaces, compiled using four different single species models: FCS alone (green lines), standard lifetime alone (yellow lines), lifetime using an intensity constraint (blue lines), and τFCS, also with an intensity constraint (red lines). Fitting with FCS alone yields a horizontal valley centered at diffusion coefficient ratio of 1, with boundaries that are consistent with Meseth *et al.* [10]. Similarly, the two lifetime models yield vertical valleys, with a slight asymmetry around the ratio of 1. This asymmetry is due to the finite pulse to pulse time window, in which the longer lifetimes of the second component do not fully decay at larger lifetime ratios. From these plots it is immediately clear that there are many experimental conditions for which either FCS or lifetime measurements alone cannot resolve two sample components. In contrast the τFCS approach greatly decreases the range of parameter space, centered at 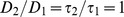, for which two species cannot be clearly resolved. This clearly indicates that τFCS improves the confidence in model selection over standard FFS methods, and also provides experimental guidelines for how different physical parameter values must be to be easily resolved experimentally using τFCS.

In Fig. 3 the τFCS chi-squared contours fall slightly outside of the single method curves since they are essentially the average of the chi-squared values for the two different methods. For example, when the two diffusion coefficients are identical and the lifetimes different the FCS fit alone will yield “good fit” with χ^2^ ∼1 while the lifetime fit alone will have a larger chi-squared value since it cannot fit the two component lifetime data as well with a single lifetime model. We note that the “good fit” for the FCS data in this example, as indicated by the chi-squared value, is in this case actually a “poor fit” for failing to resolve the presence of a second species that is contained within the sample. The τFCS chi-squared, which again basically averages the FCS and lifetime chi-squared values, thus indicates a “less poor” fit than lifetime alone. The difference in lifetime values needed to bring the τFCS chi-squared value up to the same value as lifetime fitting alone is what pushes the τFCS contour slightly outside the lifetime or FCS alone curves. This feature of these plots should be understood in terms of the regions of parameter space for which the various methods can distinguish between one- and two-component samples, and does not indicate that τFCS has lower resolution. To the contrary, as demonstrated above, the τFCS significantly enhance curve fitting resolution fitting to a two component model when compared with either method used alone.

## Discussion/Conclusions

We have introduced a new fluorescence fluctuation analysis technique, τFCS, and have demonstrated that τFCS can dramatically enhance sensitivity and resolution in fluctuation measurements, highlighted by the capability to measure concentrations, molecular brightness values, diffusion coefficients and fluorescence lifetimes from multiple molecular species even when their diffusion coefficients are very similar and one of the species makes up a small fraction of the total molecular population within the sample. Moreover, Global-τFCS removes the need to fix any of the fitting parameters during the analysis, and thus removes the need for independent measurements on isolated sample components which are often not possible to measure. The freedom from parameter assumptions and calibrations, together with the marked improvement in accuracy highlight the benefits of Global-τFCS. τFCS also has the potential to significantly enhance detection of molecular interactions. When a molecule binds to another molecule of similar molecular weight its change in diffusion coefficient is typically too small to be resolved *via* FCS analysis alone. However, it is not unusual for the fluorescence to be quenched (or dequenched) upon binding, which has an associated change in the fluorescence lifetime and thus potentially making the interaction resolvable *via* τFCS. The sensitivity of τFCS, with the possibility to resolve even a small fraction of a molecular population involved in interactions as demonstrated above, further enhances the utility of this method for resolving such interactions.

There are two important principles underlying the success of the τFCS approach. First, by using MFD it is often possible to find a contrast parameter that allows resolution of multiple sample components that are otherwise disguised in a particular measurement. For example, in the case of τFCS the fluorescence lifetime serves as the contrast parameter which detects the presence of two sample components when the FCS measurement alone would not resolve a second species. Second, and as importantly, global fitting of multiple measurement modes (e.g. FCS and lifetime) simultaneously enhances the resolution of each individual method during curve fitting. The different methods have unique dependencies on the global parameters, such as lifetime data depending linearly on the molecular brightness but the FCS data amplitude depending on the square of the molecular brightness. Thus, an analysis method that forces the fitting parameters to account for total fluorescence signal across independent measurement modes, but with common global parameters describing each, has a greatly constrained fitting parameter space and produces much better curve fitting results.

Also serving the success of this approach is the strategy to leverage all the information content of the fluorescence signal. This includes, for example, making use of the often ignored amplitude information in the lifetime data as is done in τFCS. While illustrated here for the specific case of FCS and lifetime measurements, this general principle can and should be further exploited to maximize use of the information content of the fluorescence signal. For example, measurements of molecular rotations *via* fluorescence anisotropy or spectral signatures through spectrally resolved detection could serve as further extensions of this approach. When coupled with the full global analysis approach, such additional contrast parameters will further provide important ways to enhance sensitivity and resolution for characterizing the molecular scale composition and dynamics of molecules within complex systems.

A less considered, but equally as important topic is that of determining the correct fit model, without assumptions, using data alone. The increased ability to resolve small fractions of different species provides an inherent capacity to assist in model discrimination. We have demonstrated the ability of τFCS in distinguishing the presence of a second species across a wide range of parameter combinations. The array of χ^2^ values demonstrates the increased parameter space for which τFCS can separate species and provides a metric for experimental design.

Measurements as described in this work are reasonably straight forward to implement. The theory presented is valid as long as the sample does not change in any way from one measurement to the next. The measurements thus need not in principle be acquired simultaneously, although in practice that is likely the best approach to ensure no changes in the sample from one measurement mode to the next. Some additional hardware may be required, such as time correlated single photon counting (TCSPC) electronics for lifetime measurements, but it is generally very practical to perform multiple modes of measurement at the same time, on the same instrument, for any given sample. Certainly lifetime and FCS data can be acquired simultaneously, and extending such measurements to include molecular rotations, spectra, and other parameters is a straight forward process using modern hardware. Thus, any time fluctuation measurements are contemplated, a multi-modal global analysis approach is probably also accessible – with improved accuracy and resolution as demonstrated above. It is thus likely that methods such as τFCS will eventually supersede fluctuation methods alone.

## Supporting Information

Figure S1 **Comparison of τFCS and global-τFCS analyses.** τFCS analysis (a) allows brightnesses and concentrations to be treated globally across the two data modalities due to common parameters now describing the amplitudes of both the lifetime decay and autocorrelation function. Global-τFCS (b) intrinsically retains the pair-global relationship for each pair individually, in addition to treating the lifetimes, diffusion coefficients, and brightnesses as global parameters across all pairs of the titration. Data has been normalized for visual comparison.
(TIF)

Figure S2 **Reference data set recorded using uncorrelated light to assess the systematic error in data acquisitions.** A 100 point binomial smoothed data set (black line) removes Poissonian noise while retaining the lower frequencies (red line) used for data corrections.
(TIF)

Figure S3 **The effects of molecular brightness assumptions in two component FCS analyses compared to τFCS.** Comparison of simulated data sets depicting a binary system with a diffusion coefficient ratio of 3, molecular brightness and lifetime ratios of 2 (a), and titrated across a concentration ratio of three orders of magnitude (b). Here, we have ‘calibrated’ species a and fixed the known values for D_a_, ψ_a_ and τ_a_ during all subsequent analyses. The covariant autocorrelation amplitudes require that the molecular brightnesses be held for both species using FCS analysis; therefore, we have “guessed” ψ_b_ in order to attain stable fits. Three different analyses (b) using ψ_b_ guesses below (ψ_b_  =  8 kcpsm; horizontal kites), the same as (ψ_b_  =  10 kcpsm; diamonds), and above (ψ_b_  =  12.5 kcpsm; horizontal kites) the correct molecular brightness value highlight the potential inaccuracies in two component FCS results. Across concentration ratios C_a_/C_b_ of 0.03 to 1, in which the amount of unknown species is sufficiently large, FCS analysis can distinguish the 2nd component, albeit with molecular brightness guess dependent errors. Beyond the C_a_/C_b_ of approximately 3, FCS analysis fails to identify two species and transitions into a fit result that finds two identical species of equal concentration, that of half the total. This is corroborated by the transition of the returned diffusion coefficient, D_b_, from 0.1 to 0.3 µm^2^ms^−1^ (e; all blue data points). τFCS analysis (b; gold data points) of the same titration data set, in which no assumptions or held parameters are enforced on the 2nd species, returns accurate results across the entire range, even in the case of a very small fraction of the less bright species (gold circles). τFCS also returns accurate molecular brightnesses (c) and fluorescence lifetimes (d) across the majority of the titration range, and still distinguishes the diffusion coefficient where FCS analysis fails (e; gold triangles). Data points and error bars indicate the average and standard deviation of three independent simulated data sets.
(TIF)

Figure S4 **Comparison of initial guesses in global analysis of experimental FCS data only.** To demonstrate that multi-method global analysis, here implemented as τFCS, is the key to the accuracy of the results shown in Fig. 3, we show here that global analysis of FCS data alone does not return comparable accuracy, or even stable fitting results. Shown here are experimental autocorrelation data of binary RhB and R6G mixtures, with individually calibrated parameters (a), subject to global analysis of FCS data only (no lifetime data) incorporating repeated titration data sets. Concentrations are considered local fit parameters while diffusion coefficients and molecular brightness are global. Here, the diffusion coefficient and molecular brightness of R6G have been held fixed at the correct value during fitting. Four different initial guess combinations for the diffusion coefficient and molecular brightness of RhB are shown (b-e). Some initial guesses return somewhat stable fits, albeit with inaccurate results, while other initial guesses can lead to extremely unstable fits. Data points and error bar reflect the average and standard deviation of the three different fits to the independently acquired data sets. These fitting results show that global analysis of FCS data alone cannot accurately fit the data.
(TIF)
