# Supplementary figures and images for: τFCS: Multi-Method Global Analysis Enhances Resolution and Sensitivity in Fluorescence Fluctuation Measurements
Source: PLoS One. 2014 Feb 28;9(2):e90456. doi: 10.1371/journal.pone.0090456 (PMC3938748; doi:10.1371/journal.pone.0090456)

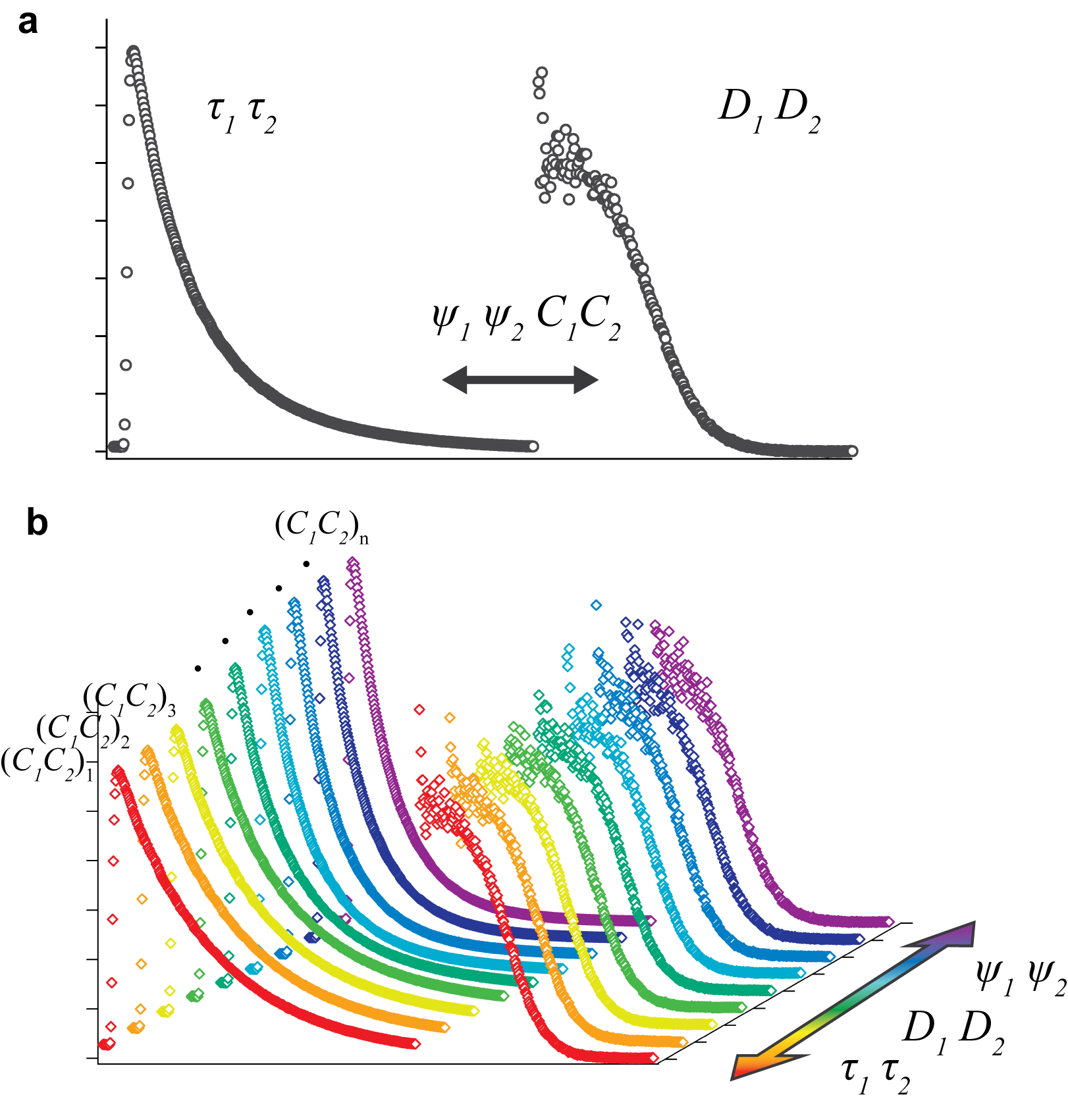

Supplement: Figure S1 — Comparison of τFCS and global-τFCS analyses. τFCS analysis (a) allows brightnesses and concentrations to be treated globally across the two data modalities due to common parameters now describing the amplitudes of both the lifetime decay and autocorrelation function. Global-τFCS (b) intrinsically retains the pair-global relationship for each pair individually, in addition to treating the lifetimes, diffusion coefficients, and brightnesses as global parameters across all pairs of the titration. Data has been normalized for visual comparison. (TIF) [file pone.0090456.s001.tif]

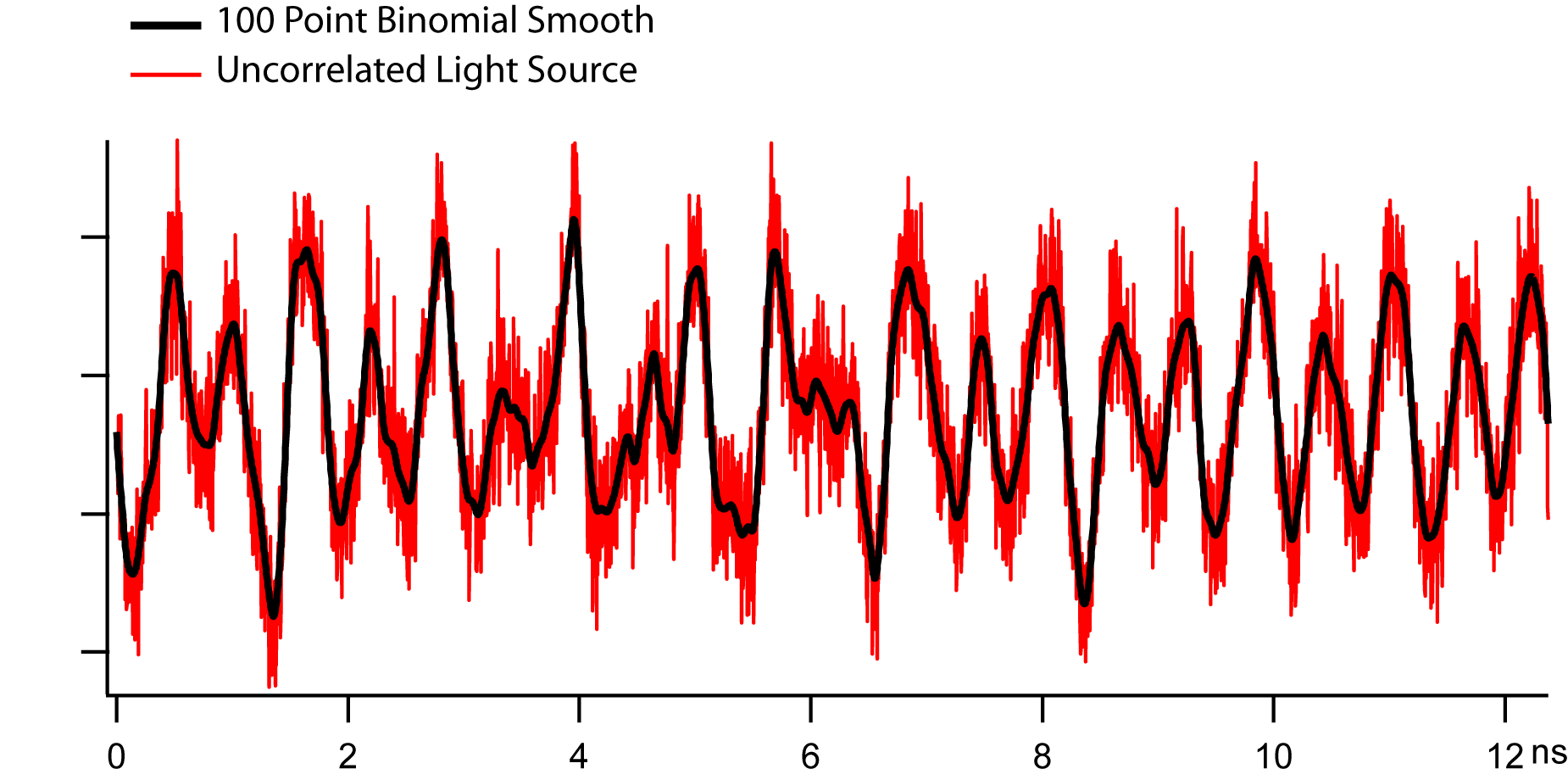

Supplement: Figure S2 — Reference data set recorded using uncorrelated light to assess the systematic error in data acquisitions. A 100 point binomial smoothed data set (black line) removes Poissonian noise while retaining the lower frequencies (red line) used for data corrections. (TIF) [file pone.0090456.s002.tif]

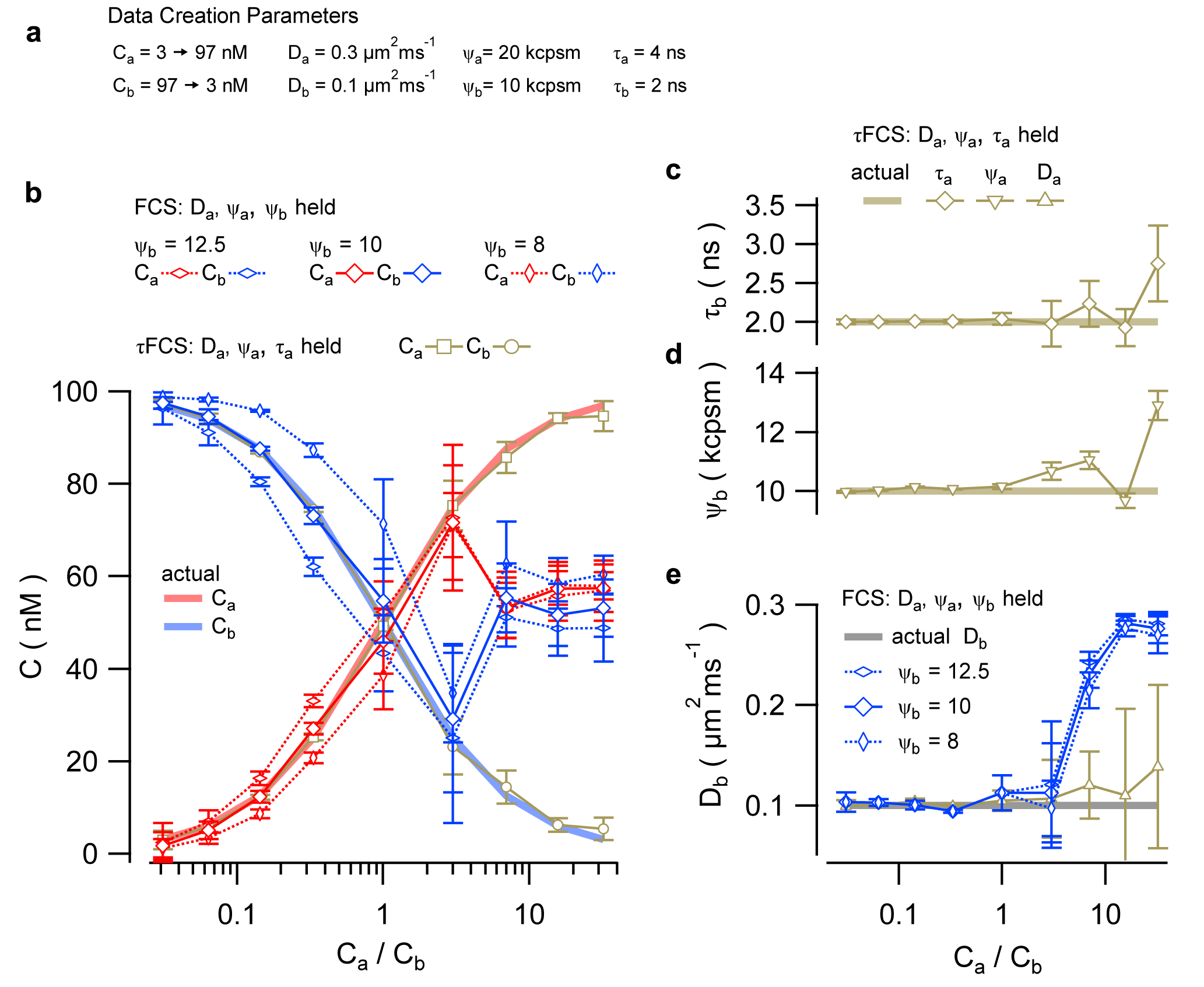

Supplement: Figure S3 — The effects of molecular brightness assumptions in two component FCS analyses compared to τFCS. Comparison of simulated data sets depicting a binary system with a diffusion coefficient ratio of 3, molecular brightness and lifetime ratios of 2 (a), and titrated across a concentration ratio of three orders of magnitude (b). Here, we have ‘calibrated’ species a and fixed the known values for Da, ψa and τa during all subsequent analyses. The covariant autocorrelation amplitudes require that the molecular brightnesses be held for both species using FCS analysis; therefore, we have “guessed” ψb in order to attain stable fits. Three different analyses (b) using ψb guesses below (ψb = 8 kcpsm; horizontal kites), the same as (ψb = 10 kcpsm; diamonds), and above (ψb = 12.5 kcpsm; horizontal kites) the correct molecular brightness value highlight the potential inaccuracies in two component FCS results. Across concentration ratios Ca/Cb of 0.03 to 1, in which the amount of unknown species is sufficiently large, FCS analysis can distinguish the 2nd component, albeit with molecular brightness guess dependent errors. Beyond the Ca/Cb of approximately 3, FCS analysis fails to identify two species and transitions into a fit result that finds two identical species of equal concentration, that of half the total. This is corroborated by the transition of the returned diffusion coefficient, Db, from 0.1 to 0.3 µm2ms−1 (e; all blue data points). τFCS analysis (b; gold data points) of the same titration data set, in which no assumptions or held parameters are enforced on the 2nd species, returns accurate results across the entire range, even in the case of a very small fraction of the less bright species (gold circles). τFCS also returns accurate molecular brightnesses (c) and fluorescence lifetimes (d) across the majority of the titration range, and still distinguishes the diffusion coefficient where FCS analysis fails (e; gold triangles). Data points and error bars indicate the average [file pone.0090456.s003.tif]

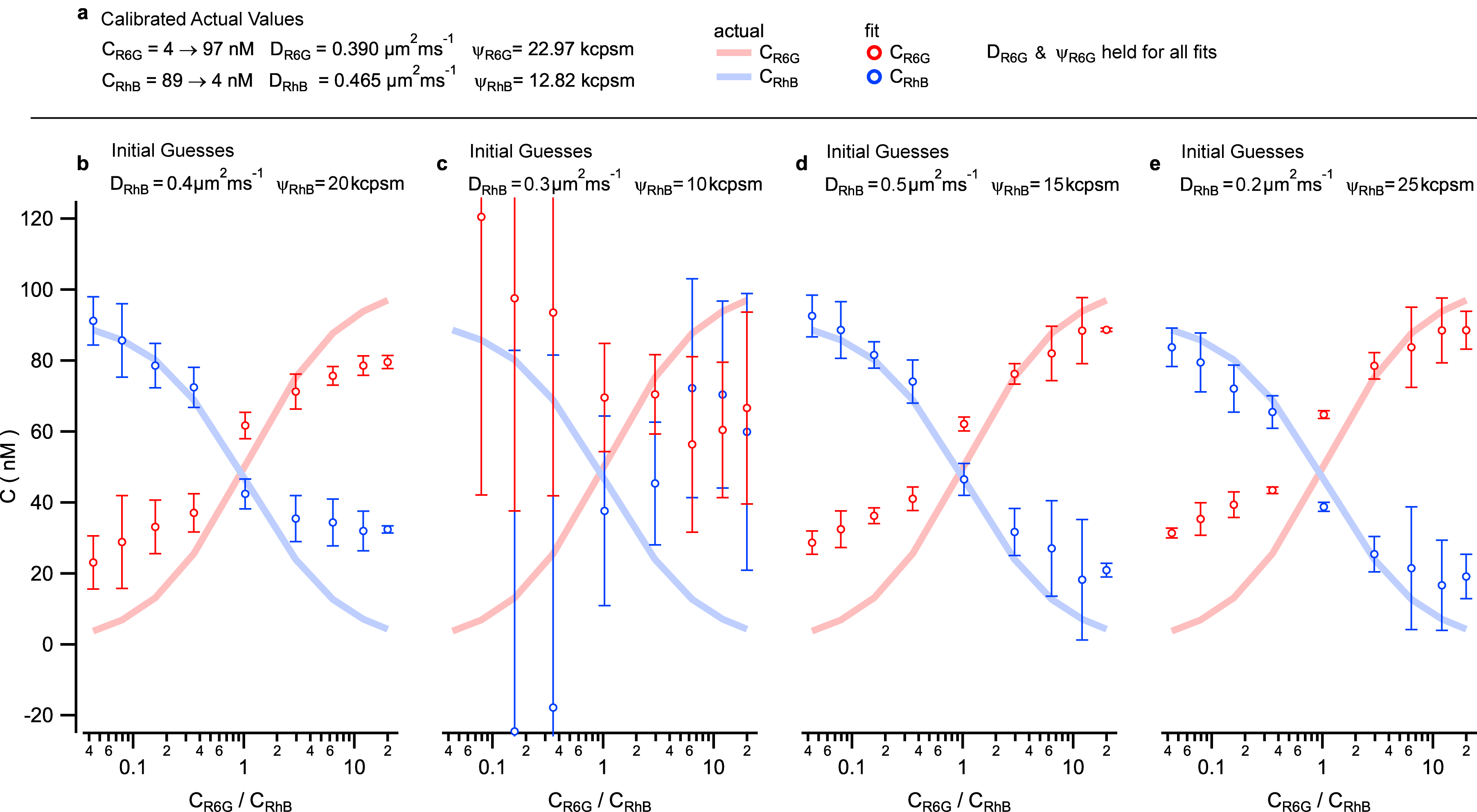

Supplement: Figure S4 — Comparison of initial guesses in global analysis of experimental FCS data only. To demonstrate that multi-method global analysis, here implemented as τFCS, is the key to the accuracy of the results shown in Fig. 3, we show here that global analysis of FCS data alone does not return comparable accuracy, or even stable fitting results. Shown here are experimental autocorrelation data of binary RhB and R6G mixtures, with individually calibrated parameters (a), subject to global analysis of FCS data only (no lifetime data) incorporating repeated titration data sets. Concentrations are considered local fit parameters while diffusion coefficients and molecular brightness are global. Here, the diffusion coefficient and molecular brightness of R6G have been held fixed at the correct value during fitting. Four different initial guess combinations for the diffusion coefficient and molecular brightness of RhB are shown (b-e). Some initial guesses return somewhat stable fits, albeit with inaccurate results, while other initial guesses can lead to extremely unstable fits. Data points and error bar reflect the average and standard deviation of the three different fits to the independently acquired data sets. These fitting results show that global analysis of FCS data alone cannot accurately fit the data. (TIF) [file pone.0090456.s004.tif]
